# Supplementary material for: Selective Targeting and Enhanced Photodynamic Inactivation of Methicillin-Resistant Staphylococcus aureus (MRSA) by a Decacationic Vancomycin–Mesochlorin Conjugate
Source: Antibiotics (Basel). 2025 Sep 28;14(10):978. doi: 10.3390/antibiotics14100978 (PMC12561113; doi:10.3390/antibiotics14100978)
Supplement: Supplementary file 1 [file antibiotics-14-00978-s001.zip › antibiotics-3847358-supplementary.pdf]

Selective Targeting and Enhanced Photodynamic Inactivation of Methicillin-Resistant Staphylococcus aureus (MRSA) by a Decacationic Vancomycin–Mesochlorin Conjugate

He Yin [1,†], Xiaojing Liu [2,3,†], Min Wang [1], Ying Wang [2], Tianhong Dai [2,*], Long Y. Chiang [1,*]

[1] Department of Chemistry, University of Massachusetts Lowell, Lowell, MA 01854, USA

[2] Wellman Center for Photomedicine, Massachusetts General Hospital, Harvard Medical School, Boston, MA 02114, USA

[3] Institute of Photomedicine, Shanghai Skin Disease Hospital, School of Medicine, Tongji University, Shanghai, 200443, China

* Correspondence: tdai@mgh.harvard.edu (T.D.); LongYong_Chiang@uml.edu (L.Y.C.)

† These authors contributed equally to this work.

Supporting Information

Preparation of 15b-methyl-13a,17c-di[aminopropylpolyoxyethylene]-chlorin e_6 (Chl_{pd}-EG_n, LC37, nPS). Preparation method of **LC37** follows those reported previously with slight modification [30]. Briefly, to the solution of pheophytin a (0.30 g, 0.35 mmol) and bis(3-aminopropyl) terminated oligo(ethylene glycol) (EG_n, M_w 1500, 1.5 g, 1.5 mmol) in CH_2Cl_2 (20 mL), was added trifluoroacetic acid (0.3 mL, 3.9 mmol) slowly under N_2. The mixture was stirred at r.t. for 10 h followed by the solvent evaporation to give raw products. They were purified by column chromatography (neutral alumina) using dichloromethane–acetone (1:2) as the eluent to afford green solids of the precursor intermediate Chl-EG_n in a yield of 82% (1.02 g). A portion of this intermediate (0.20 g, 0.056 mmol) was dissolved in H_2O and treated with $PdCl_2$ (0.011 g, 0.06 mmol) for 8.0 h, followed by reacting with hydroiodic acid (HI, 0.016 g, 0.12 mmol) to afford a crude product. It was concentrated by rotary-evaporation for water removal. The solid was quickly washed by cold H_2O to remove a trace amount of residual $PdCl_2$ and HI and further dried to give Chl_{pd}-EG_n (**LC37**, **nPS**) in a nearly quantitative yield. Spectroscopic data: ^{1}H NMR (500 MHz, DMSO-d_6, ppm) δ 9.57 (s, 1H), 9.32 (s, 1H), 8.35 (s, 1H), 8.00 (1H), 6.24 (1H), 6.03 (1H), 4.67–4.01 (8H), 3.82–2.99 (m, br, ~140H), 2.62–2.28 (m, 4H), 1.67–1.51(4H), 1.31–1.18 (2H). FT-IR (KBr) ν_{max} 3362 (m), 2955 (s), 2918 (s), 2848 (s), 1737 (s), 1698 (s), 1621 (s),

1542 (m), 1498 (w), 1450 (m), 1384 (s), 1345 (m), 1296 (w), 1257 (w), 1221 (w), 1195 (w), 1123 (vs), 1092 (vs), 1033 (s), 796 (m), 725 (w), 668 (w), 616 (m), and 593 (m); UV–vis (DMF, 1.0 × 10^{-5} M) λ_{max} (ε) 406 (3.88 × 10^4), 502 (6.94 × 10^3), 536 (5.79 × 10^3), 610 (4.16 × 10^3), and 668 nm (1.09 × 10^4 L·mol^{-1}·cm^{-1}). For Fig. 2Aa in DMF (2.0 × 10^{-5} M) λ_{max} (ε) 409 (0.88), 505 (0.1), 535 (0.07), 610 (0.07), and 667 nm (0.34 a.u.); FL (DMF, 2.0 × 10^{-5} M, λ_{ex} 410 nm) $\lambda_{em,max}$ 468 (1.46 × 10^3) and 674 nm (4.79 × 10^4 counts/s).

Preparation of 15b-methyl-13a,17c-di[N,N′,N,N,N,N-hexapropyl-panta(aminoethylene)] amide-[Pd^{+2}]chlorin e$_6$-deca(quaternary methyl-ammonium iodide) (Chl$_{Pd}$-N$_{10}^+$, LC38$^+$, dcPS). Preparation method follows those reported previously with slight modifications [30]. Briefly, to the solution of pheophytin *a* (0.30 g, 0.35 mmol) and *N,N′,N,N,N,N*-hexapropyl-penta(aminoethylene)amine (HPAA, 0.36 g, 0.72 mmol) in dichloromethane (20 mL), was added trifluoroacetic acid (0.23 mL, 3.0 mmol) slowly under N_2. The mixture was stirred at r.t. for 10 h followed by solvent evaporation to give raw products, which were purified by column chromatography (neutral alumina) using the eluent of dichloromethane–acetone (1:1) to afford green solids of the precursor intermediate Chl-N$_{10}$ (**LC38**) in a yield of 80% (0.43 g). Spectroscopic data: FT-IR (KBr) ν_{max} 2989 (w), 2976 (m), 2938 (w), 2880 (w), 1738 (s), 1733 (s), 1663 (s), 1656(m), 1630 (s), 1624 (m), 1590 (w), 1542 (m), 1510 (m), 1458 (s), 1435 (m), 1389 (m), 1381 (m), 1350 (m), 1235 (m), 1179 (m), 1211 (w), 1041 (m), 985 (m), 960 (m), 893 (w), 845 (w), 815 (w), 784 (w), 766 (w), 740 (m), 674 (m), and 605 (m) cm^{-1}; UV–vis (DMF, 1.0 × 10^{-5} M) λ_{max} (ε) 414 (4.01 × 10^4), 510 (5.26 × 10^3), 538 (4.42 × 10^3), 566 (2.71 × 10^3), 610 (3.39 × 10^3), and 670 nm (1.50 × 10^4 L·mol^{-1}·cm^{-1}) or λ_{max} (ε) (DMF, 2.0 × 10^{-5} M) 410 (0.87), 509 (0.11), 540 (0.08), 612 (0.07), and 670 nm (0.38 a.u.); ^{1}H NMR (500 MHz, $CDCl_3$, ppm) δ 9.60 (s, 1H, east pole of chlorin ring), 9.48 (s, 1H, north pole of chlorin ring), 8.63 (s, 1H, west pole of chlorin ring), 8.17 (s, 1H), 8.04 (d, 1H, secondary olefin proton), 6.31 (1H, primary olefin proton), 6.20 (1H, primary olefin proton), 4.0–2.75 (centered at δ 3.04, 80H, >N-CH_2–), 2.50 (2H), 1.73 (30H), and 0.94 (m, 36H, –CH_3).

In the subsequent step, the intermediate Chl-N$_{10}$ (**LC38**, 0.20 g, 0.13 mmol) was dissolved in DMF and treated with $Pd(OAc)_2$·2H2O (0.032 g, 0.14 mmol) for 8.0 h, followed by reacting with CH_3I (excess) at 45 °C for 24 h. During the solvent removal process by rotary-evaporation, an excessive amount of CH_3I, was also eliminated. The crude products were washed by chloroform and ether to afford Chl$_{Pd}$-N$_{10}^+$ (**LC38$^+$**) as green solids in a yield of 92% (366 mg). Spectroscopic data: FT-IR (KBr) ν_{max} 2997 (w), 2966 (m), 2928 (w),

2874 (w), 2846 (w), 1732 (s), 1663 (s), 1630 (m), 1624 (m), 1586 (w), 1549 (m), 1501 (m), 1469 (s), 1431 (m), 1386 (m), 1376 (m), 1355 (m), 1231 (m), 1179 (m), 1211 (w), 1062 (w), 1039 (m), 989 (m), 969 (m), 892 (w), 845 (w), 815 (w), 785 (w), 760 (w), 740 (m), 671 (m), and 602 (m) cm^{-1}; UV–vis (DMF, 1.0 × 10^{-5} M) λ_{max} (ε) 412 (4.22 × 10^4), 506 (5.20 × 10^3), 534 (4.53 × 10^3), 560 (2.71 × 10^3), 607 (3.44 × 10^3), and 666 nm (1.42 × 10^4 $L{\cdot}mol^{-1}{\cdot}cm^{-1}$). For Fig. 2Ab in DMF (2.0 × 10^{-5} M) λ_{max} (ε) 408 (0.92), 505 (0.11), 536 (0.08), 609 (0.07), and 666 nm (0.36 a.u.); FL (DMF, 2.0 × 10^{-5} M, λ_{ex} 410 nm) $\lambda_{em,max}$ 466 (2.43 × 10^3) and 673 nm (5.77 × 10^4 counts/s); ^{1}H NMR (500 MHz, DMSO-d_6, ppm) δ 9.55 (s, 1H), 9.30 (s, 1H), 8.33 (s, 1H), 7.99 (1H), 6.21 (1H), 6.02 (1H), 3.12–4.48 (m, br, 110H), 2.54 (2H), 2.47 (2H), 2.35 (2H), 1.60–1.91 (m, br, 30H), and 0.96 (m, 36H).

Preparation of 3a-hydroxy-3b-aminoethylene-aminoPEGlycolated vancomycin-15b-methyl-13a,17c-di[N,N′,N,N,N,N-hexapropyl-panta(aminoethylene)]amide-meso-chlorin-deca(quaternary methyl-ammonium iodide) (VCMe-mChl$_{Pd}$-N_{10}^+, LC40e, Abx-dcPS). Same chemistry was used as major route to prepare **LC40e**$^+$ [30]. Step A. To the solution Chl-N_{10} (**LC38**, 0.32 g, 0.21 mmol) in THF–H_2O (3.0:1.0, v/v) was added *m*-chloroperoxybenzoic acid (*m*CPBA, 0.039 g, 0.22 mmol) to epoxidate the olefin bond (C_{3a}=C_{3b}, Fig. 1) connected on the chlorin core moiety. It was followed by the addition of ethylenediamine (0.015 g, 0.25 mmol) to effect the oxirane ring-opening of epoxide moiety in the presence of a di(*n*-butyl)tin(IV) dilaurate (T12, 150 μL) catalyst and triethylamine (150 μL, 1.1 mmol) at 60 °C for 3.0 h. The reaction progress was monitored by thin-layer chromatography (neutral alumina) using the eluent of CH_2Cl_2–acetone (1:1). A small amount of excessive ethylenediamine and Et_3N were removed along with the solvent removal using rotary evaporator to afford a precursor intermediate of 3a-hydroxy-3b-aminoethyleneamino-*m*Chl-N_{10}.

Step B. To the solution of oligo(ethylene glycol) diglycidyl ether (M_n ~500, 0.10 g, 0.2 mmol) in THF–H_2O (3.0:0.7, v/v, 37 mL) was added di(*n*-butyl)tin(IV) dilaurate (T12, 100 μL) catalyst and triethylamine (150 μL, 1.1 mmol). The mixture was stirred at 60 °C for 20 min. Vancomycin hydrochloride (1.0 equiv., 0.30 g, 0.21 mmol) in H_2O (3.0 mL) was then added slowly in a period of 2.0 h to ensure the monoaddition reaction in a dilute solution. The mixture was stirred for another 1.0 h, followed by the addition of 3a-hydroxy-3b-aminoethylene-amino-*m*Chl-N_{10} (from the step A) in THF–H_2O (3.0:0.7, v/v, 3.7 mL) to effect the reaction with the remaining glycidyl group at 60 °C for 3.0 h. Raw products

obtained were dissolved in dil. HCl to remove the insoluble 4-chlorobenzoic acid generated during the reaction. Resulting precursor intermediate was obtained as green solids after neutralization by saturated K_2CO_3 in DMF. The solids were further extracted by methanol to remove an excessive amount of vancomycin and K_2CO_3. Subsequent treatment of resulting products with $Pd(OAc)_2$ (0.050 g, 0.22 mmol), followed by CH_3I at 45 °C in DMF affords VCMe-*m*Chl_{Pd}-N_{10}^+ (**LC40e**, **Abx-dcPS**) in a yield of 88% (0.93 g). Spectroscopic data: ^{1}H NMR (500 MHz, D_2O, ppm) δ 9.83 (s, 1H, east pole of mesochlorin ring), 9.73 (s, 1H, north pole of mesochlorin ring), 9.08 (s, 1H, west pole of mesochlorin ring), 7.66 (1H, VCM), 7.58 – 7.49 (m, 2H, VCM), 7.24 (1H, VCM), 7.08 (1H, VCM), 6.89 (1H, VCM), 6.48 (1H, VCM), 6.44 (1H, VCM), 5.48 (2H, VCM), 5.38 (2H, VCM), 5.28 (1H, VCM), 4.21–3.78 (5H, VCM), 3.62 (24H, EG_n) 3.52–2.79 (centered at 3.39, 110H, >N-CH_2-CH_2-CH_3, −CH_3 from CH_3I and VCM), 2.64 (s, 3H, −CH_3 next to secondary amine of VCM), 2.01 (2H, VCM), 1.82–1.32 (28H, >N-CH_2-CH_2-CH_3), 1.10 (s, 3H, VCM), and 0.94 (m, 42H, >N-CH_2-CH_2-CH_3). FT-IR (KBr) ν_{max} 2984 (m), 2980 (w), 2964 (w), 2946 (w), 2874 (w), 2858 (w), 1746 (s), 1679 (s), 1657 (s), 1618 (m), 1599 (m), 1560 (m), 1508 (m), 1465 (m), 1454 (m), 1426 (m), 1400 (m), 1379 (m), 1370 (m), 1360 (m), 1345 (m), 1314 (w), 1297 (m), 1249 (m), 1235 (m), 1179 (w), 1116 (s), 1110 (s), 1061 (m), 1029 (m), 991 (m), 960 (m), 952 (m), 892 (w), 886 (m), 848 (w), 826 (w), 769 (w), 740 (m), 712 (m), and 610 (m) cm^{-1}; UV-vis (DMF, 1.0×10^{-5} M) λ_{max} (ε) 284 (2.64×10^4), 410 (2.22×10^4), 512 (3.54×10^3), 556 (2.64×10^3), 580 (2.33×10^3), and 677 nm (2.38×10^3 $L{\cdot}mol^{-1}{\cdot}cm^{-1}$). For Fig. 2Ac in DMF ($2.0 \times 10^{-5}$ M) λ_{max} (ε) 408 (0.94), 501 (0.14), and 665 nm (0.18 a.u.); FL (DMF, 2.0×10^{-5} M, λ_{ex} 410 nm) $\lambda_{em,max}$ 467 (5.23×10^3) and 676 nm (5.75×10^4 counts/s); Owing to the multiple molecular segment weight distributions of EG_n oligomer (M_n ~500), MALDI-TOF spectrum was not obtainable. For the similar reason, in addition to the variation of the counter salt as either I^- or I_3^-, elemental analyses data were not accountable. Therefore, ^{1}H NMR spectroscopic correlations of LC40 to those of LC38, LC39 and the parent vancomycin, having the closely related structural moieties and the identical synthetic methods applied, were the primary characterization methods [30].

Preparation of tetrasodium α,α'-(anthracene-9,10-diyl)bis(methylmalonic acid salt) (ABMA). *α,α'*-(anthracene-9,10-diyl)bis(methylmalonic acid) (100 mg, 0.24 mmol) was suspended in H_2O (5.0 mL), with sodium hydroxide (0.05 g), followed by ultrasonication for a period of 5.0 min to give a clear solution. The tetrasodium salt product was precipitated upon the addition of ethanol. The solids were washed three times with ethanol and dried in vacuo to yield ABMA in 81% (97 mg). Spectroscopic data: FT-IR (KBr)

νmax 3446 (vs, water peak), 2951 (w), 2923 (w), 2892 (w), 2844 (w), 1592 (vs), 1423 (m), 1339 (m), 1317 (s), 892 (m), 815 (w), 757 (m), 695 (w), 628 (w), 594 (w), 517 (w) cm−1. UV–vis (H2O, 1.0 × 10−5 M) λmax 327 (shoulder band, ε = 9.62 × 105), 344 (ε = 2.54 × 106), 361 (ε = 5.65 × 106), 380 (ε = 9.38 × 106) and 402 (ε = 9.12 × 106 cm2/mol) nm; 1H NMR (500 MHz, D2O, ppm) δ 8.46 (d, 4H), 7.62 (dd, 4H), 4.16 (d, 4H), and 3.56 (t, 2H); 13C NMR (500 MHz, D2O, ppm) δ 179.00 (4C), 132.37 (2C), 129.40 (4C), 125.53 (2C), 125.41 (2C), 125.36 (2C), 125.30 (2C), 59.77 (2C), and 28.29 (2C).

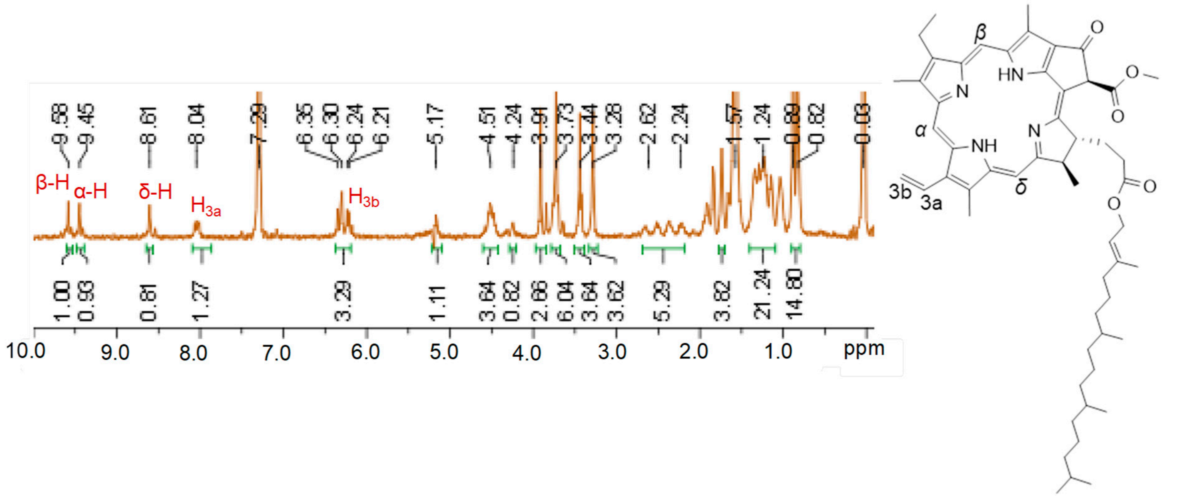

Figure S1. ^{1}H NMR spectrum of pheophytin *a* (Phe) in $CDCl_3$.

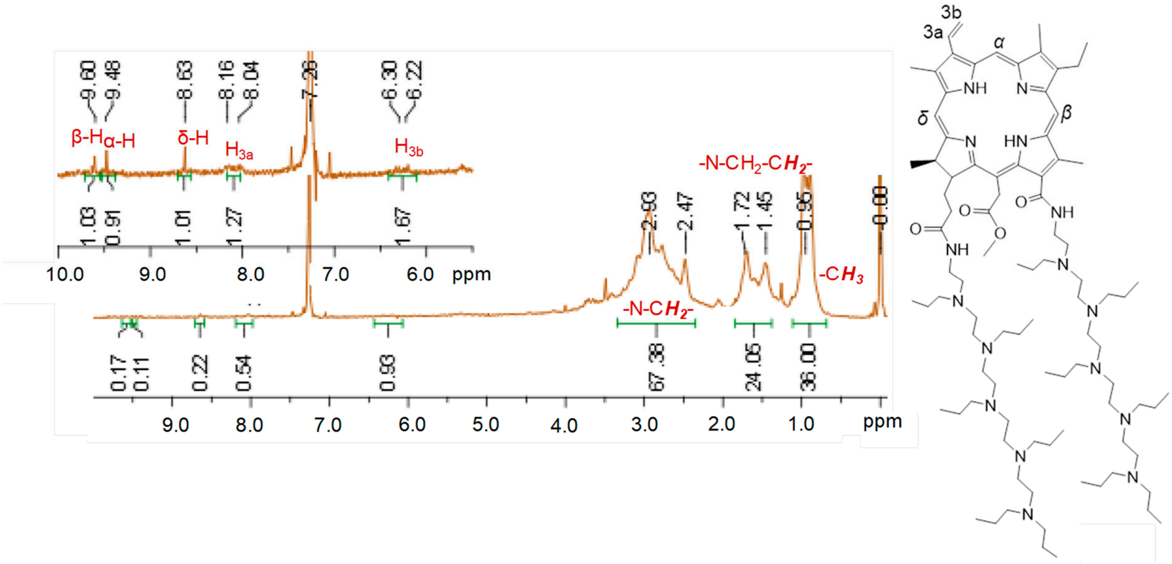

Figure S2. ^{1}H NMR spectrum of non-metalated intermediate of LC38 as Chl-N_{10} in $CDCl_3$. It showed the approximate retention in chemical shift value of three pheophytin-*a* ring protons *α*-H, *β*-H, and *δ*–H at *δ* 8.5–9.6, as indicated. Vinyl proton (H_{3a} and H_{3b}) peaks in the spectrum centered at *δ* 8.16 and 6.25, respectively, consistent with one olefin bond at C_{3a}–C_{3b} carbons. The overall proton integration in the region of *δ* 0.5–4.0 matches well with the corresponding addition of two new neutral N_6-arms of Chl-N_{10} with the assigned proton peaks given.

We selected the integration of $H_δ$ located on $C_δ$ or $H_β$ on $C_β$ of chlorin core at *δ* 8.63 or 9.60, respectively, as the reference of one-proton count (1.0). Theoretically, integration of two types of protons of each N_5-amide arm with the chemical shift at *δ* 2.5–3.25 (–N-CH_2–, aminomethylene protons) and *δ* 0.65–1.08 (–CH_3, end-group methyl protons) should

give proton counts of 32(H) and 18(H), respectively, in a ratio of 1.78. By taking the measured proton counts integrated over two chemical shift ranges at δ 2.5–3.25 and δ 0.65–1.08 as 67.38 and 24.05, respectively, as the base, we then deducted all other types of non-N_5-amide associated methyl and methylene protons appearing and overlapping in the same chemical shift regions. It resulted in adjusted values of 62.4 and 34.1 for two N_5-amide-armed Chl-N_{10}. These values corresponded well with a proportional ratio of –N-**C**H_2–/–**C**H_3 protons of Chl-N_{10}, giving a roughly good agreement with the number of N_5-amide arms of 1.85−1.95. These results can be regarded as a good consistency with the structural assignment of the compound after functional modification from the basic chlorin core moiety.

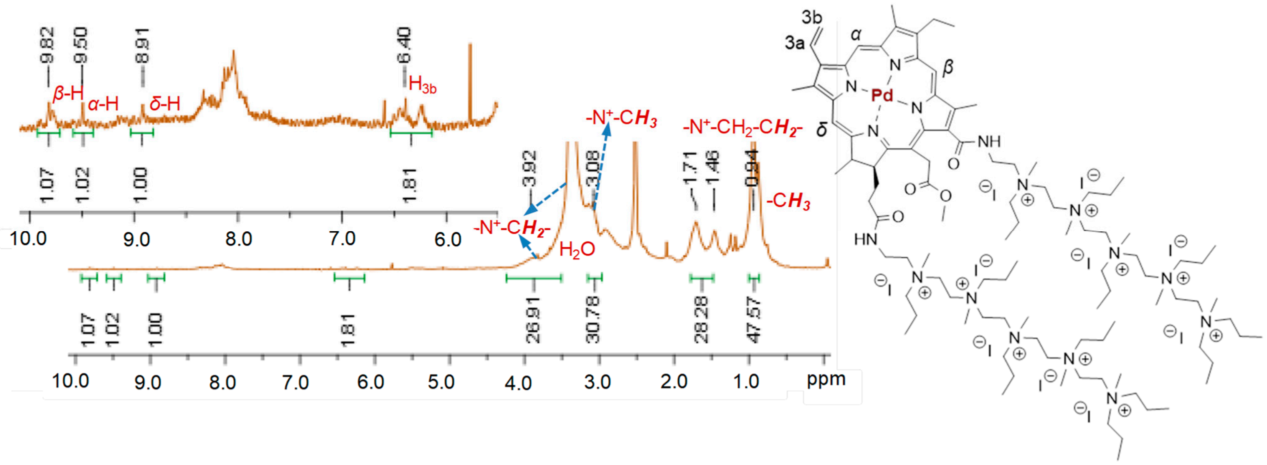

Figure S3. ^{1}H NMR spectrum of photosensitizer Chl_{pd}-N_{10}^+ (LC38$^+$) in DMSO-d_6.

To verify the degree of quaternization, we compared ^{1}H NMR spectra between non-metalated neutral Chl-N_{10} (LC38, in $CDCl_3$, Figure S3) and methyl quaternized metalated decacationic Chl_{pd}-N_{10}^+ (LC38$^+$, in DMSO-d_6, Figure S4) showed nearly quantitative conversion using methyl iodide as the quaternization agent. The major difference is between the chemical shift of -N-**C**H_2- protons. In neutral compound, The precursor Chl-N_{10} compound displayed the chemical shift of methylene protons (−**C**H_2-N<) next to tertiary amines at δ 2.5–3.4. Upon quaternization by CH_3I, the chemical shift of these methylene protons downfield-shifted to δ 3.1–4.1 leaving the region of δ 2.6–3.0 with minimum proton bands in the spectrum. Corresponding proton peak assignments were marked by red. These shifts of proton peaks were indicative of approximately full chemical conversion to decacationic states as Chl_{pd}-N_{10}^+. Meanwhile, a new sharp singlet peak of methyl (N^+-

CH_3) protons next to the quaternary amine was detected at δ 3.09, as marked by red above.

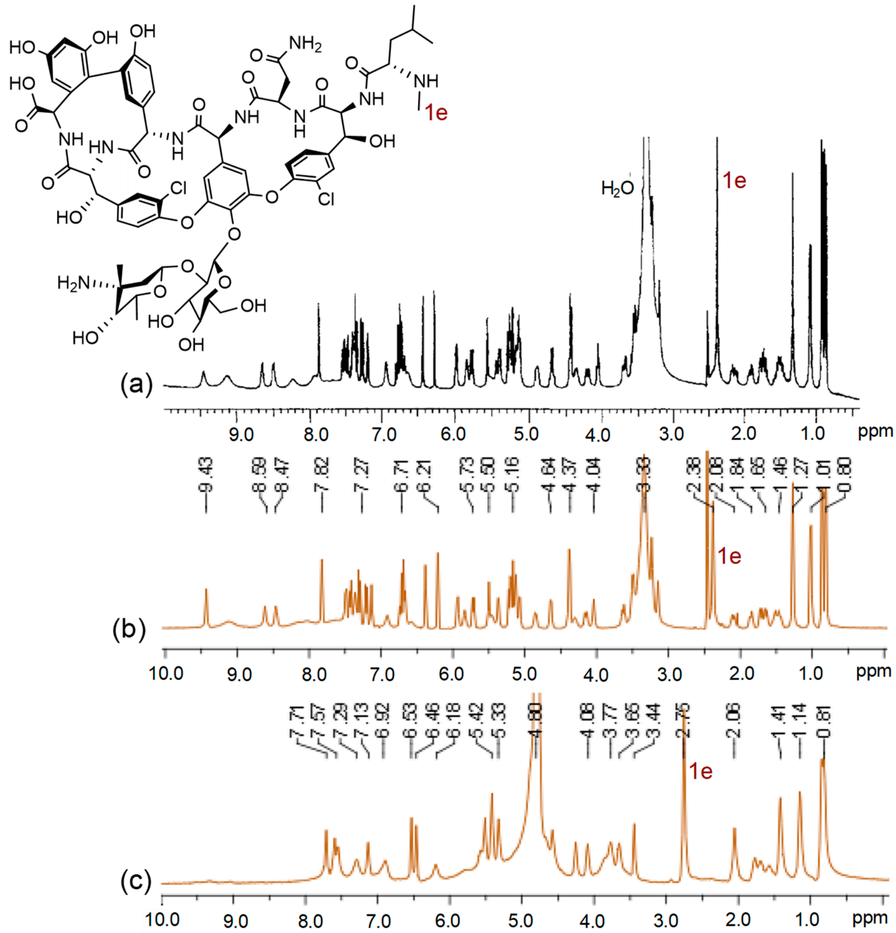

Figure S4. ^{1}H NMR spectrum of (a) vancomycin from the reference literature [Pearce, C.; Williams, D. Complete assignment of the ^{13}C NMR spectrum of vancomycin, *J. Chem. Soc. Perkin Trans*. **1995**, *2*, 153–157.] in DMSO-d_6, (b) our vancomycin (VCM) in DMSO-d_6, and (c) our vancomycin in D_2O for comparison of the proton peak shifts due to solvent effects.

^{1}H NMR comparison of vancomycin in different deuterium solvents. This comparison was necessary since LC40e is water-soluble. Its ^{1}H NMR spectrum was collected in D_2O. Fig. (c) will allow us to correlate all peak chemical shift positions to the VCM moiety of LC40e. Apparently, the chemical shift of $-CH_3$ (marked as **1e** in brown) proton next to the secondary amine in VCM is located at δ 2.76 in D_2O instead of δ 2.38 in DMSO-d_6. In addition, certain peak position shifts due to the solvent effect were detected.

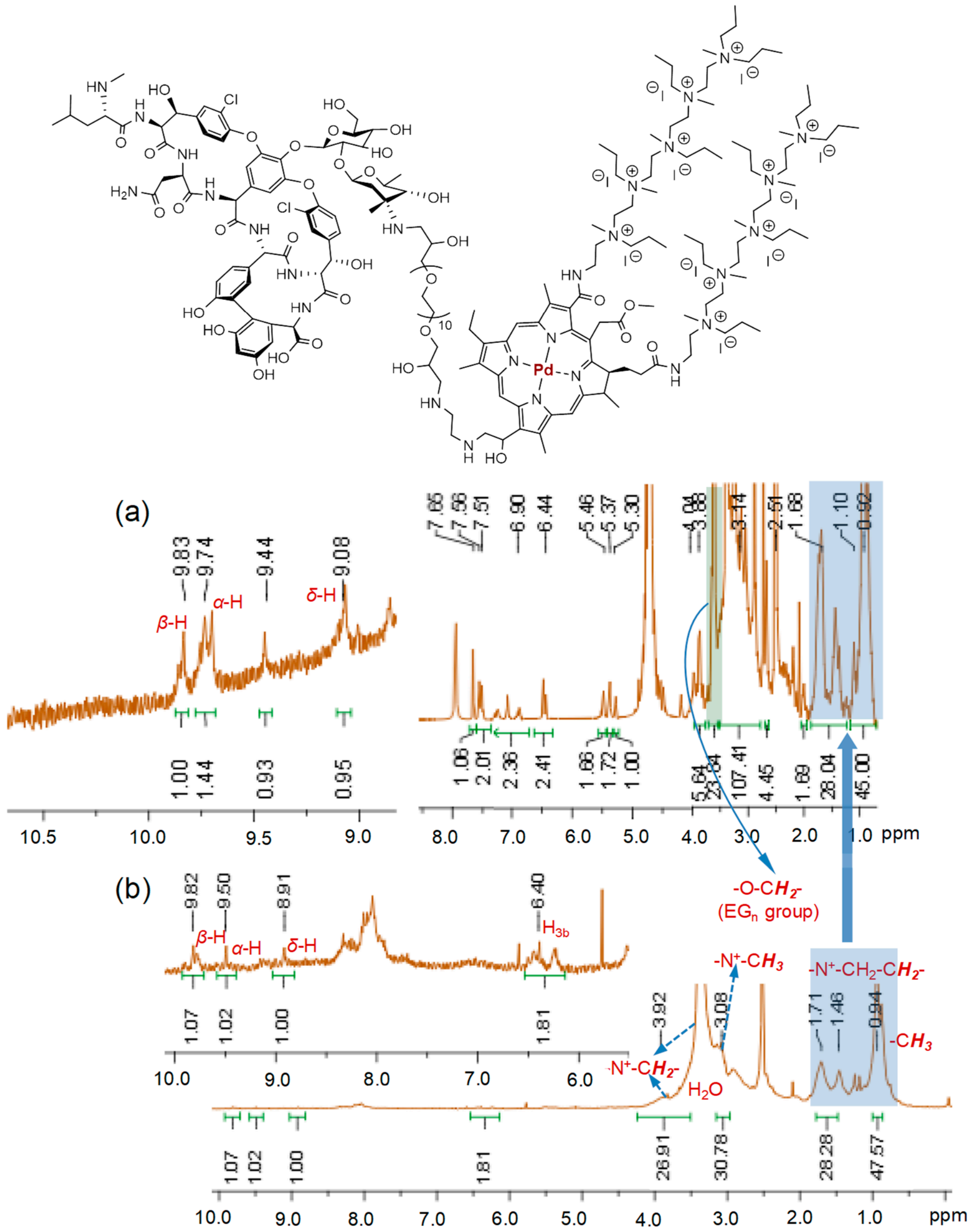

Figure S5. ^{1}H NMR spectrum of photosensitizers (a) VCMe-*m*Chl$_{Pd}$-N$_{10}^{+}$ (LC40e$^+$) in D_2O and (b) LC38$^+$ in DMSO-*d*$_6$, showing the combination of chlorin ring moiety proton peaks at δ 9.0–9.85, vancomycin moiety proton peaks at δ 5.0–8.1, oligo(ethylene glycol) (EG$_n$) protons at δ 3.7, and all propyl group protons at δ 0.7–1.8, with the former two matching with those of Figures S4 and S5c, respectively. Several key proton groups were assigned in the spectrum by red.

By using the VCM spectrum in D_2O (Figure S5c) as the reference for comparison, the proton peak profiles of VCM moiety of LC40e at δ 5.0–8.2 are almost identical to each other, indicating successful covalent attachment of VCM to the chlorin-ring moiety. Whereas

three protons as H_δ located on C_δ, H_β on C_β, and H_α on C_α of mesochlorin-ring core region of decacationic $LC38^+$ at δ 8.91, 9.82, and 9.50 (Figure S6b), respectively, was correlated to the corresponding proton peaks of mesochlorin-ring core region of decacationic $LC40e^+$ at δ 9.08, 9.83, and 9.50 (the inset of Figure S6a), respectively. By considering the difficulty to detect these hydrophobic protons in D_2O, not in contact with the aqueous solvent, we were still able to locate roughly the same three chemical shift positions for H_α, H_β, and H_δ in the spectrum by amplification. These two major components in the structure of LC40e co-exist in one spectrum provided the evidence of their covalent bonding together. Regarding to the oligo(ethylene glycol) (EG_n) linker located between the mesochlorin-ring and VCM moieties was also detected by its new ethylene glycol proton peaks at δ 3.7 in the spectrum of Figure S5 a as comparing that of Figure S6b. In conclusion, the ^1H NMR spectrum of $LC40e^+$ was found to be consistent with its chemical structure.

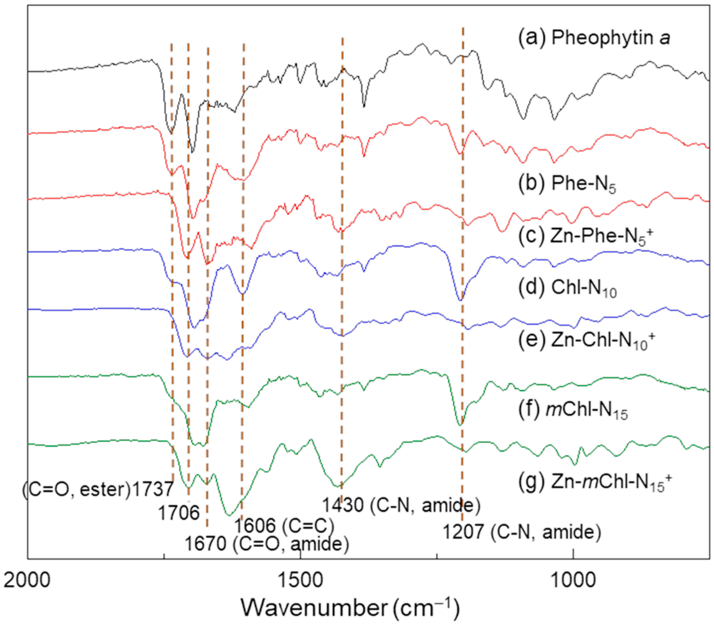

Figure S6. Infrared spectroscopic comparison between the precursor compounds and products upon functional group changes from (a) pheophytin *a* (Phe), (b) Phe-N_5 (LC35), (c) pentacationic Phe_{Zn}-N_5^+ (LC35$^+$), (d) Chl-N_{10} (LC38), (e) decacationic Chl_{Pd}-N_{10}^+ (LC38$^+$), (f) *m*Chl-N_{15} (LC36), to (g) pentadecacationic *m*Chl_{Zn}-N_{15}^+ (LC36$^+$).

Various spectroscopic methods were applied for the characterization of all compounds with the corresponding data included in the experimental section. Since all methyl, ethyl side groups, and ring protons of pentacationic Phe_{Zn}-N_5^+ (LC35$^+$), decacationic Chl_{Pd}-N_{10}^+ (LC38$^+$), and pentadecacationic *m*Chl_{Zn}-N_{15}^+ (LC36$^+$) remained identical to those of the parent pheophytin *a* (Phe) core, therefore, all spectroscopic analyses were based on the comparison with the precursor compound to match with the consistent functional group changes. Accordingly, progressive changes of three infrared absorption bands (Figure S6) of these three compounds at 1670 [–**C**(=**O**)-NH–], 1606 (–**C=C**–), and 1430/1207 [–**C**(=O)-(**N**–H)–] cm^{-1} were evaluated as the indicator of structural modification. Intensity increases of the former band going from pheophytin *a* (Phe Figure S6a) to Phe-N_5 (LC35, Figure S6b) and pentacationic Phe_{Zn}-N_5^+ (LC35$^+$, one amide arm, Figure S6c), Chl-N_{10} (LC38, Figure S6d) and decacationic Chl_{Zn}-N_{10}^+ (LC38$^+$, two amide arms, Figure S6e), and then *m*Chl-N_{15} (LC36, Figure S6f) and pentadecacationic *m*Chl_{Zn}-N_{15}^+ (LC36$^+$, three amide arms, Figure S6g) were apparently consistent with an increase of pentacationic N_5^+-amide arm(s). Similar phenomena were also detected on the latter 1430/1207-band as the absorption of amide C-N functional groups.
